# Apoptosis, Necrosis, and Necroptosis in the Gut and Intestinal Homeostasis

**DOI:** 10.1155/2015/250762

**Published:** 2015-09-21

**Authors:** Anna Negroni, Salvatore Cucchiara, Laura Stronati

**Affiliations:** ^1^Department of Radiobiology and Human Health, ENEA, Via Anguillarese 301, 00123 Rome, Italy; ^2^Department of Pediatrics and Infantile Neuropsychiatry, Pediatric Gastroenterology and Liver Unit, Sapienza University of Rome, Viale Regina Elena 324, 00161 Rome, Italy

## Abstract

Intestinal epithelial cells (IECs) form a physiochemical barrier that separates the intestinal lumen from the host's internal milieu and is critical for electrolyte passage, nutrient absorption, and interaction with commensal microbiota. Moreover, IECs are strongly involved in the intestinal mucosal inflammatory response as well as in mucosal innate and adaptive immune responses. Cell death in the intestinal barrier is finely controlled, since alterations may lead to severe disorders, including inflammatory diseases. The emerging picture indicates that intestinal epithelial cell death is strictly related to the maintenance of tissue homeostasis. This review is focused on previous reports on different forms of cell death in intestinal epithelium.

## 1. Introduction

Intestinal homeostasis depends on complex interactions between microbiota, intestinal epithelium, and host immune system. Diverse regulatory mechanisms cooperate to maintain intestinal homeostasis, and a failure in these pathways may lead to chronic inflammatory disorders [1–3].

The intestinal epithelium represents a huge surface area that is lined by a monolayer of intestinal epithelial cells (IECs), which serve as a barrier to luminal microbes, while also allowing the absorption of water and nutrients essential to life, sensing both beneficial and harmful microbes, and inducing and modulating immune responses [4]. To fulfill such diverse functions, the intestinal epithelium comprises several specialized cell types, divided into two main groups: the absorptive cells, represented by enterocytes, and the secretory cells including Paneth cells, goblet cells, and enteroendocrine cells [5–8]. These subsets of IECs are functionally different and essential to maintain intestinal homeostasis by separating the intestinal lumen from the underlying lamina propria and by controlling the crosstalk between microbiota and subjacent immune cells.

Maintaining barrier function and commensal composition in healthy intestine is also ensured by a basal activation of pattern recognition receptors (PRRs), as Toll-like receptor (TLR) [9], nucleotide oligomerization domain- (NOD-) like receptor (NLR) [10, 11], and retinoic acid-inducible gene- (RIG-) I-like receptor (RLR) [12] families, able to detect and control various microbial structures. PRRs can activate specific inflammatory transduction signaling that are intimately interconnected with different cell death pathways [13, 14], establishing a relationship between host defense mechanisms and cell death.

Intestinal epithelial homeostasis is maintained by a strict equilibrium between cell proliferation in the crypt and cell shedding from the villus tip. In the large and small bowel, differentiated enterocytes are removed constantly and replaced by new cells originated by undifferentiated adult intestinal stem cells, located in the third or fourth position counted from the base of the crypt [15]. These new cells migrate from the base of the crypt to the apical zone of the intestine undergoing maturation. The epithelial layer displays a strict balance between cell proliferation and cell death in order to maintain the intestinal barrier [16].

**Figure 1:**
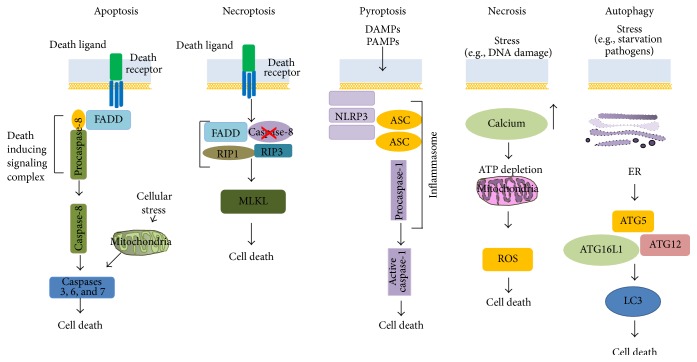
Cell death pathways. Apoptosis, necroptosis, and pyroptosis are programmed forms of cell death, while necrosis represents an unregulated cell death. Autophagy is a survival pathway that if it is excessive or uncontrolled, it promotes cell death. Fas-associated protein with a death domain (FADD); receptor-interacting interacting protein 1 (RIP1); receptor-interacting interacting protein (RIP3); mixed lineage kinase domain-like (MLKL); danger-associated molecular patterns (DAMPs); pathogen-associated molecular patterns (PAMPs); nod-like receptor family, pyrin domain containing (NLRP)3; apoptosis-associated speck-like protein containing a caspase recruitment domain (ASC); autophagy-related genes (ATG); autophagy related 16-like 1 (ATG16L1); light chain 3 (LC3II); ER: endoplasmic reticulum.

In this review we will describe the relationship between the main forms of epithelial cell death, apoptosis, necrosis and necroptosis, and the intestinal epithelium during gut homeostasis and inflammation. Moreover, two secondary forms of cell death will be shown (Figure 1).

## 2. Cell Death and Intestinal Epithelium

Cell death is a crucial process for tissue development and equilibrium to eliminate superfluous, damaged, or aged cells and represents a key for the homeostasis reestablishment after an acute or chronic insult, limiting the propagation of the inflammatory stimuli to prevent tissue loss of function [17]. This is of particular importance for the gastrointestinal tract, since the intestinal epithelium undergoes continuous and rapid self-renewal, while it is permanently exposed to a plethora of antigens and potential pathogens which are present in the food and in the microbial flora. As a consequence, epithelial cell renewal and cell death need to be tightly regulated because inappropriate cell death responses inexorably lead to the development of diseases, like inflammatory disorders and cancer [18, 19]. Historically, cell death has been divided in unregulated forms, such as necrosis, and programmed forms, such as apoptosis and necroptosis.

Traditionally, necrosis has been described as a passive and uncontrolled process, initiated by external factors such as ischemia-reperfusion, toxins, viral, and bacterial infections and is characterized by a rapid breakdown of the cell membrane, resulting in the release of intracellular compounds into the extracellular space with activation of the immune system [20, 21].

Apoptosis is a process relying on caspase activation that when it is excessive, it may may impair the epithelial barrier, leading to severe gut pathology [16, 22, 23]. Apoptosis has long been considered the only form of regulated cell death, while the existence of additional forms of controlled cell death is now well established [24, 25]. The latter can be triggered independently of apoptosis induction or as back-up safety mechanisms when the apoptotic machinery does not operate properly, as a result of genetic mutations or chemical or microbial inhibition.

Necroptosis is a recently identified form of programmed cell death that is, differently from apoptosis, negatively regulated by caspases and depending on the kinase activity of receptor-interacting proteins (RIP) [26, 27]. Necroptosis shows morphological features similar to necrosis, but, as apoptosis, is strictly regulated by a multiprotein platform. It is characterized by a rapid membrane breakdown, resulting in the release of intracellular compounds, that is, Damage-Associated Molecular Patterns (DAMPs), such as high-mobility group box 1 (HMGB1) protein, heat shock proteins, DNA, and RNA, that activate PRRs to further promote an inflammatory response [25, 28].

Given the complex structure of the intestinal epithelium, proliferation, differentiation, and cell death must be tightly controlled. Excessive cell death might result in a breakdown of the intestinal barrier with subsequent uncontrolled access of bacteria into the gut wall and inflammation [1]. On the contrary, resistance to cell death is believed to be a driving force of tumor development in the gut [28].

## 3. Apoptosis 

Apoptosis can be initiated by a wide variety of stimuli including DNA damage, nutrient deficiency, endoplasmic reticulum (ER) stress, growth factor withdrawal, heat shock, developmental cues, and ligation of death-receptors on the cell surface [22, 23] through the activation of apoptotic caspases [23, 29].

Two sites of epithelial cell death have been described along the length of the villus: the first takes place in the crypt at the level of the stem and early transit cells and is sometimes referred to as “spontaneous” apoptosis and the second at the villus tip or to the surface epithelial cuff in the colon, where epithelial cells, after travelling from the crypt base, differentiate and then die from anoikis [30]. The latter is a special form of programmed cell death induced in anchorage-dependent cells after detachment from their matrix [31, 32]. Mechanisms control this process are still unsettled [30]. Indeed, cell detachment is suggested to coincide with morphological hallmarks of apoptotic cell death, but a clear causal relationship between cell detachment and apoptosis has not yet been proven [31–33].

A number of studies implicate that cell shedding is actively regulated and involves the proapoptotic molecule caspase-3 [34, 35] and that the block of caspases inhibits the tumor necrosis factor- (TNF-) induced cell shedding [35]. Furthermore, it has been reported that TNF or lipopolysaccharide (LPS) stimulation increases apoptosis and consequent cell shedding and is associated with barrier loss [36]. Differently, several studies on mouse models did not support the apoptotic hallmark of shedding epithelial cells. Studies on mice null for proapoptotic molecules, caspase-3, caspase-8, and Fas-associated protein with a death domain (FADD) did not show any morphological alterations in the development of the gastrointestinal epithelium, suggesting that apoptosis is not required for intestinal turnover [37, 38]. Furthermore, experimental data obtained with the use of Necrostatin-1, a chemical inhibitor of the kinase receptor interacting protein (RIP)1, showed that murine enterocyte shedding in the small intestine is mostly associated with a nonapoptotic programmed cell death, mediated by RIP1 [39]. Recent studies with RIP1 knock-out mice showed that the loss of caspase-8 or TNFR1 completely prevented the intestinal pathology, suggesting a RIP1 essential role in protecting the intestinal epithelia from apoptosis [40–42].

The most convincing hypothesis is that epithelial cell shedding might be a passive process induced by high density of cells in constrained spaces at the villus tip and, thus, the shedding-associated cell death might be a consequence rather than a cause of shedding [43].

Alongside with intestinal cell shedding, patterns of spontaneous apoptosis, p53 mediated, have been described within the crypt region, but with a different regulation between the large and small intestine [44, 45]. Indeed, it concerns stem cell region in the small intestine but it is rarely found in the colonic crypts. Accordingly, the antiapoptotic gene Bcl-2 (B-cell CLL/lymphoma 2) [45] is barely expressed in the small intestine and strongly expressed at the base of the colonic crypts. Interestingly, differences in Bcl-2 expression and cell death regulation can account for the variability in tumor prevalence between the small and large intestines [46]. Two other studies on mouse models showed that Bcl-2 and Bax-null mice displayed similar levels of spontaneous apoptosis in small intestinal crypts compared to their wild-type strains [47, 48].

Altogether these findings highlight that the role of apoptosis in physiology of healthy gut is still controversial.

## 4. Apoptosis and Intestinal Inflammation

An increasing body of evidence suggests that apoptotic signaling may promote inflammatory processes by releasing extracellular vesicles and various chemokines, which may potentially recruit and activate immune cells [49].

Although the role of apoptosis in the structural integrity of the gut is still controversial, there is little doubt that dysregulated or excessive apoptosis can lead to severe gut disorders. Accordingly, several studies reported that mice with elevated apoptosis in the intestinal epithelium more likely develop gut inflammation [46, 50, 51].

Mice lacking nuclear factor kappa B essential modulator (NEMO) in intestinal epithelial cells (IECs) developed spontaneous colitis shortly after the birth due to excessive TNF-dependent apoptosis, followed by epithelial barrier breakdown and translocation of bacteria into the bowel wall. The inhibition of TNF signaling avoided the development of colitis [52]. Moreover, silencing of other members of the NF-*κ*B pathway, REL-A, transforming growth factor-activated kinase (TAK)1 or both I*κ*B kinase (IKK)1 and IKK2, in IECs increased murine susceptibility to spontaneous colitis, providing a strong link among NF-*κ*B activity, epithelial apoptosis, and intestinal inflammation [53–55].

Furthermore, mice with IEC-specific deletion of the transcription factor Stat3 showed an increased sensitivity to apoptosis upon treatment with dextran sodium sulfate (DSS) [56]. Similarly, XBP1 knock-out mice developed a spontaneous enteritis associated to Paneth cell dysfunction and increased apoptosis of intestinal barrier cells [57].

A dysregulated apoptosis has also been suggested to play a role in the pathogenesis of human inflammatory bowel disease (IBD), including Crohn's disease (CD) and ulcerative colitis (UC). It was reported that an excessive cell shedding and barrier loss in IBD patients in remission predict a disease relapse [58]. Moreover, various apoptotic bodies were found in colonic biopsies routinely taken from patients with active UC, especially in those who required surgery compared with those only treated with medication, suggesting a correlation between IEC apoptosis and disease severity [59]. A twofold increased apoptosis was observed in colonic samples of CD patients compared to controls. Interestingly, levels of apoptosis returned to control levels when patients underwent anti-TNF therapy, suggesting a role of TNF in epithelial cell death [60, 61].

T cells in the intestinal mucosa of IBD patients were found to be resistant to multiple apoptotic signals, showing defects in the control of programmed cell death, thus suggesting a possible mechanism to explain why inflammation is resilient to resolution in IBD patients [62–64].

Cell death in the intestinal epithelium seems also to be regulated by bacterial communities. Increased apoptosis has been demonstrated in patients infected with human pathogens, including* Salmonella*,* Shigella*, enteropathogenic* Escherichia coli*, human immunodeficiency virus type 1,* Helicobacter pylori*, and* Cryptosporidium parvum* [65–67].

In conclusion, although these studies show a relationship between IBD pathogenesis and altered apoptosis, it is still unclear if the latter is a primary event or a secondary to inflammation.

## 5. Necrosis 

Necrosis is considered as an accidental and uncontrolled cell death, frequently associated to disorders such as ischemia-reperfusion (IR) injury, neurodegeneration, intestinal ischemia, and infarction [20, 21, 68, 69]. During necrosis, cell and organelles swell and break down with subsequent release of cellular content into the microenvironment, causing the inflammatory response. Common players in necrotic cell death are calcium, which causes mitochondrial calcium overload, bioenergetics effects as well as activation of proteases and phospholipases, and reactive oxygen species (ROS), which produce mitochondrial dysfunction, ion balance deregulation, and loss of membrane integrity [69–71]. Membrane destabilization is also mediated by additional factors, such as acid-sphingomyelinase (ASM), phospholipase A(2) (PLA(2)), and calpains [72]. It has been shown that necrotic cells release immunomodulatory factors causing the activation of the immune response [21, 70, 71].

Necrotic cells have been observed in the colonic epithelium of CD patients, within inflamed as well as uninflamed areas, suggesting that increased necrosis might be a primary, rather than a secondary, mechanism for the development of the disease [73]. Furthermore, the histological analysis revealed, in subjects with IBD, the presence of necrotic intestinal epithelial cells, within the crypts, even during the inactive phase of the disease, implying that necrosis occurs more frequently than expected [74]. In a recent study, the necrosis of Paneth cells in the terminal ileum has been linked to the pathogenesis of IBD [37]. Necrosis as well dysfunction of Paneth cells could explain the decreased production of antimicrobial peptides in IBD [75].

However, whether IEC necrosis normally occurs in the mucosa of the gastrointestinal tract or is strictly associated to inflammation remains still elusive.

Necrosis is also believed to play a fundamental role in infectious gastrointestinal diseases caused by pathogens, since it has been showed that it can be driven by several cytotoxic bacteria [76, 77].

## 6. Necroptosis

Recently, a new caspase-independent mode of programmed cell death, termed necroptosis, has been identified [31, 78, 79], also in the intestinal epithelium [23, 37, 38].

Although necroptosis shows morphological features similar to necrosis, it is highly regulated by an intracellular protein platform, largely overlapping with that of apoptosis [25, 26, 79–81]. However, while apoptosis depends on caspase activation, necroptosis is negatively regulated by caspases and needs the intervention of the kinase RIP3 that is thought to be a necroptosis key mediator [82–85]. It is conceivable that when the apoptotic caspases fail to be activated, then cells undergo necroptosis as an alternative death pathway [79, 84].

Different endogenous or exogenous stimuli, such as the TNF, ligation of Fas, or the engagement of innate immunity sensors [79, 83], may trigger the necroptosis by activating RIP3 [82–85].

**Figure 2:**
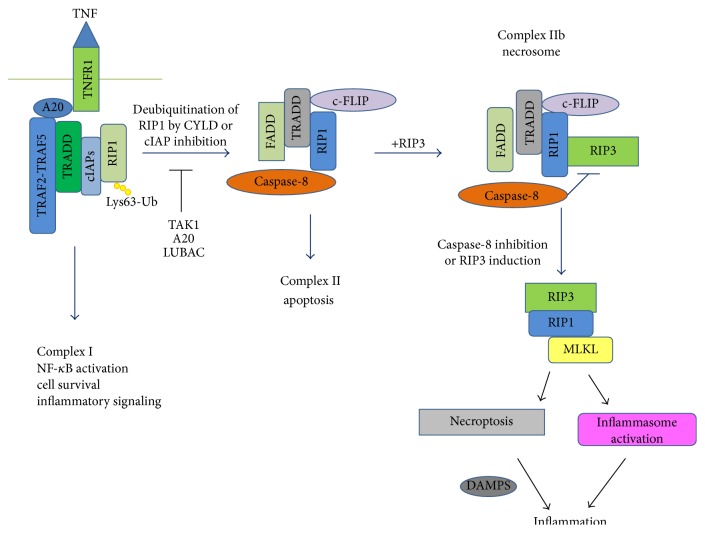
TNF-induced programmed cell death pathways. Cell death is induced by various stimuli that are recognized by specific sensors. These receptors recruit the complex I protein platform (TRADD, RIP1, TRAF2, and cIAP1/2), activating the inflammatory response and survival. Deubiquitination of RIP1 induces complex II (FADD and caspase-8), activating the apoptotic pathway. Inhibition of caspase-8 and increase of RIP3 expression induces complex IIb (necrosome), activating the necroptotic pathway. TNF receptor 1 (TNFR1); TNFR-associated death domain (TRADD); receptor-interacting interacting protein 1 (RIP1); receptor-interacting interacting protein (RIP3); TNFR-associated factor 2 (TRAF2); inhibitor of apoptosis proteins 1 and 2 (cIAP1/2); linear ubiquitin chain assembly complex (LUBAC); Lys63-linked polyubiquitination (Lys63-Ub); LUBAC; TAB-transforming growth factor-activated kinase 1 (TAK1); cylindromatosis (CYLD); Fas-associated protein with a death domain (FADD); mixed lineage kinase domain-like (MLKL); Dynamin-related protein (Drp1); dendritic cells (DCs); Damage-Associated Molecular Patterns (DAMPS).

Currently, the most informative studies of necroptosis pathway derive from systems that use TNF as a trigger [37, 85, 86], inducing three interrelated signaling pathways, initiated by distinct scaffolding complexes named complex I, complex II, and complex IIb, leading the cell to three different outcomes [23–25]. Upon binding to its receptor, the TNF receptor 1 (TNFR1), the TNF forms the membrane-bound complex I, consisting of the TNF receptor-associated proteins with a death domain (TRADD), the TNFR-associated factor 2 (TRAF2), and RIP1 [86, 87]. After deubiquitination that destabilizes complex I, RIP1 recruits the Fas-associated protein with a death domain (FADD) and caspase-8 to form complex II, the classical extrinsic apoptotic pathway [87]. If present, RIP3 forms the complex IIb, also known as necrosome [88]. The inhibition of the caspase-8 and the simultaneous increase of RIP3 promote the activity of the necrosome resulting in necroptosis [42, 84, 89] (Figure 2).

At present, little is known about the events occurring downstream RIP1 and RIP3 that regulate necroptosis. It is suggested that the RIP homotypic interaction motif (RHIM) on RIP3 and RIP1 allows their interaction and is required for necroptosis induction. Afterwards, RIP1 and RIP3 assemble into cytosolic filamentous structures beta-amyloids, although it is unclear whether they represent a real signaling platform or a postevent accumulation of the two interacting kinases [21, 25, 90]. The RIP1-RIP3 complex drives the RIP3 autophosphorylation which, in turn, phosphorylates the mixed lineage kinase domain-like (MLKL), leading to membrane permeabilization [88, 91–96]. The occurrence of a cross-regulation between apoptotic and necroptotic pathways to maintain cell homeostasis has been suggested; accordingly, necroptosis may act as an emergency back-up death pathway when the apoptotic cascade is impaired [79]. Several in vivo studies demonstrated a role of FADD and caspase-8 to regulate necroptosis during embryonic development, since excessive necroptosis caused the death of the embryos [97, 98].

Recent papers on knock-out mice showed kinase-independent RIP1 functions regulating homeostasis and preventing inflammation in barrier tissues by inhibiting epithelial cell apoptosis and necroptosis [40, 41]. Indeed, IEC-specific RIP1 deletion caused apoptosis, villus atrophy, loss of goblet and Paneth cells, and premature death in mice. Epithelial FADD ablation inhibited IEC apoptosis and prevented the premature death of mice with IEC-specific RIP1 knockout. However, mice lacking both RIP1 and the apoptotic factor FADD in IECs displayed RIP3-dependent necroptosis, Paneth cell loss, and focal erosive inflammatory lesions in the colon [41].

## 7. Necroptosis and Intestinal Inflammation

Necroptosis, similarly to necrosis, is characterized by the release into the extracellular milieu of immunogenic cytosol content, including alarmins and Damage-Associated Molecular Patterns (DAMPS), that lead to the activation of PRRs, for example, TLRs triggering inflammation [99–102]. Necroptosis has been suggested to play a role in the pathogenesis of several inflammatory disorders, including IBD [23, 27, 28, 37, 89].

Caspase-8 and FADD null mice in IECs exhibited chronic inflammation characterized by extensive epithelial necroptosis with a marked reduction in Paneth cell number and consequent decrease of antimicrobial peptide production [36–38]. However, it is still uncertain whether necroptosis promotes inflammation or if the latter depends on the specific depletion of Paneth cells [37, 75]. High levels of RIP3 have also been shown in both adult [37] and pediatric [103] CD patients. However, necroptotic triggering factors remain unclear in both mice and humans [101].

## 8. Novelties in Cell Death: Pyroptosis and Autophagy

Pyroptosis, initially described in immune cells during antimicrobial response [104], is a caspase-1 or caspase-11-dependent regulated type of cell death that plays a central role in inflammation and immunity [105–107].

Caspase-1-dependent pyroptosis [107, 108], has been better described than caspase-11-dependent pyroptosis [109]. Pyroptosis is commenced by the interaction between pathogen-associated molecular patterns (PAMPs) and intracellular PRRs leading to the formation of a multiprotein complex called inflammasome [110] which is composed of dimers of the adaptor protein apoptosis-associated speck-like protein containing a caspase recruitment domain (ASC). A role of RIP3 in activation of inflammasome has also been reported [111]. Four subfamilies of inflammasome have been defined: nucleotide-binding domain, leucine-rich-repeat-containing family, pyrin domain-containing (NLRP)1, NLRP3, NLR family CARD domain-containing protein 4 (NLRC4), and absent in melanoma 2 (AIM2). Currently, the best characterized inflammasome is NLRP3 [112]. Stimulation with specific microbial and endogenous molecules triggers inflammasome assembly and caspase-1 activation that leads to the secretion of proinflammatory cytokines, including interleukin-1*β* (IL-1*β*) or interleukin-18 (IL-18) [110]. In the gut, the inflammasome activation has been largely associated with NOD-like receptors and DAMPs that start the enzymatic cascade triggering the inflammatory process [113–115]. The caspase-1 inflammasome is crucial in maintaining intestinal homeostasis. Indeed, mice deficient in NLRP3, NLRP6, NLRC4, ASC, caspase-1, and IL-18 are susceptible to DSS-induced colitis [116]. Interestingly, mice deficient in caspase-11 are also susceptible to DSS-induced colitis, but independently of IL-1*β* and IL-18 secretion, suggesting the existence of a mechanism distinct from classical inflammasome function in the gut [117, 118]. Indeed, a protective mechanism of caspase-11-dependent pyroptosis in the intestine has been postulated, whose deficiency might drive the disease [119].

Autophagy represents a homeostatic cellular mechanism for the turnover of organelles and proteins, through a lysosome-dependent degradation pathway [120]. During starvation or other stress conditions, autophagy facilitates cell survival through the recycling of metabolic precursors, while excessive or uncontrolled autophagy promotes cell death and morbidity [121]. The concept of autophagic cell death is based on observations of increased morphological features (e.g., accumulation of autophagic vesicles) in dying cells [122]. Targeted cytoplasmic constituents are included in a double-membraned vesicle known as autophagosome, which is then fused with a lysosome and its cargo degraded and recycled [123]. Some authors suggested that autophagy might play a role in regulating the outcome of other programmed cell death forms as apoptosis, necroptosis, and pyroptosis [114, 121, 124].

Autophagy-related genes (ATG) are essential regulators of autophagy in development and most other stages of adult life in mice [125]. Homozygous genetic knockouts of most ATG genes (e.g., ATG3, ATG 5, ATG 6, ATG 7, ATG 9, and ATG16L1) in mice are developmentally lethal [125].

Autophagy contributes to the maintenance of intestinal homeostasis, being implicated in nutrient sensing and turnover, as in the control of glucose and amino-acids level and in the recycling of lipids and some micronutrients as iron [126]. A role of autophagy in the modulation of intestinal microbiota and response to bacterial infection has also been recognized [127]. Actually, in intestinal cells, autophagy operates as part of the cell-intrinsic innate immunity program to restrict bacterial replication and dissemination [127].

The identification of the single nucleotide polymorphism (SNP) of the autophagy-related 16-like 1 (ATG16L1) gene, increasing the susceptibility to CD, established a link between autophagy and IBD [128, 129]. Recently, a defective autophagy has been also related to the activation of inflammasome, induction of pyroptosis, and increased susceptibility to colitis in mouse models [13, 130].

## 9. Conclusions

Preserving the integrity of the epithelial barrier by regulating the rate of cell death is considered crucial for maintaining the intestinal homeostasis. Failure of barrier functions, due to an unregulated or excessive cell death, leads to the entry of noxious agents and aberrant stimulation of the intestinal immune system. It is worth noting that the same immune pathways that mediate pathogen recognition and inflammation may themselves trigger cell death, emphasizing the role of the latter in host defense. Besides, the identification of the cross-regulatory relationship between different forms of cell death and their intersection with the inflammatory response are fundamental issues to understand their involvement in the development of human intestinal diseases.
